# Risk of smoking cessation treatment dropout: a cohort to help (re)think care

**DOI:** 10.1590/0034-7167-2023-0537

**Published:** 2024-08-30

**Authors:** Janaina Pereira da Silva, Adriana Ignácio de Pádua, Ruan Víctor dos Santos Silva, Felipe Lima dos Santos, Poliana Silva de Oliveira, Priscila Norié Araujo-Betetti, Joris Thievenaz, Cinira Magali Fortuna

**Affiliations:** I Universidade de São Paulo. Ribeirão Preto, São Paulo, Brazil; II Université Paris Est-Créteil. Créteil, Île de France, France; III Universidade de São Paulo, Hospital das Clínicas. Ribeirão Preto, São Paulo, Brazil; IV CY Cergy Paris Université. Gennevilliers, Île de France, France

**Keywords:** Tobacco Use Disorder, Tobacco Control, Patient Dropout, Public Health, Public Health Nursing, Tabaquismo, Control del Tabaco, Pacientes Desistentes del Tratamiento, Salud Pública, Enfermería en Salud Pública

## Abstract

**Objectives:**

to evaluate the relative risk of smoking cessation treatment dropout during its intensive phase.

**Methods:**

a retrospective and quantitative cohort study was developed from the electronic medical records of individuals who started smoking cessation treatment between 2015 and 2019 at a specialty clinic in a city in the interior of São Paulo, Brazil. The relative risk of dropping out of treatment was calculated using the Poisson regression model.

**Results:**

it was observed that out of the 396 (100.0%) individuals who started the treatment, 109 (27.5%) abandoned it before the end of the intensive phase. For each one-year increase in age, the risk of dropping out of smoking cessation treatment decreased by an average of 2%.

**Conclusions:**

the risk of dropping out of smoking cessation treatment is higher among younger individuals. It is necessary to rethink the care offered to younger adults to promote the continuity of treatment.

## RESUMEN

**Objetivos:**

evaluar el riesgo relativo de abandono del tratamiento para dejar de fumar durante su fase intensiva.

**Métodos:**

un estudio de cohorte retrospectivo y cuantitativo se desarrolló a partir de los registros médicos electrónicos de individuos que comenzaron el tratamiento para dejar de fumar entre 2015 y 2019 en una clínica de especialidades en una ciudad del interior de São Paulo, Brasil. El riesgo relativo de abandonar el tratamiento se calculó utilizando el modelo de regresión de Poisson.

**Resultados:**

se observó que, de los 396 (100.0%) individuos que iniciaron el tratamiento, 109 (27.5%) lo abandonaron antes del final de la fase intensiva. Por cada aumento de un año en la edad, el riesgo de abandonar el tratamiento para dejar de fumar disminuyó en un promedio del 2%.

**Conclusiones:**

el riesgo de abandonar el tratamiento para dejar de fumar es mayor entre los individuos más jóvenes. Es necesario repensar el cuidado ofrecido a los adultos más jóvenes para promover la continuidad del tratamiento.

## INTRODUCTION

The 2030 Agenda is an initiative by the United Nations (UN) that proposes achieving 17 Sustainable Development Goals (SDGs), forming an action plan for people, the planet, and prosperity through 169 targets. Goal number 3 of the Agenda is to “ensure healthy lives and promote well-being for all at all ages.” This goal comprises 13 targets, two of which are related to smoking: “strengthen the implementation of the World Health Organization Framework Convention on Tobacco Control (WHO FCTC) in all countries” and “reduce by one-third premature mortality from non-communicable diseases through prevention and treatment, and promote mental health and well-being”^(1)^.

Smoking is a public health issue that negatively impacts the population’s health due to its direct relationship with the development of cardiovascular, pulmonary, and oncological diseases^(2)^. Individuals in this condition incur personal costs (expenses for purchasing cigarettes, treatment of diseases caused by smoking, inhalation of tobacco smoke) as well as costs to those around them (passive smokers).

In Brazil, the National Tobacco Control Policy (PNCT) is the guiding axis of the WHO FCTC. Despite being threatened by the interference of the production chain, the PNCT has managed to establish impactful measures, such as increasing taxes and prices on tobacco products and restricting advertising that encourages its use^(3)^. These measures have reduced the estimated prevalence of smokers in Brazil from 18.2% in 2008 to 12.6% in 2019^(4)^. While many countries are still in the early stages of developing measures aimed at controlling smoking, Brazil is dedicated to expanding its treatment network and consolidating its care line^(5)^.

Article 14 of the WHO FCTC urges the implementation of measures to facilitate tobacco cessation, including treatment for smoking. Intensive approaches for smoking treatment involve structured therapeutic counseling and may or may not include pharmacotherapy. These approaches include an initial individual clinical evaluation and four structured sessions, preferably weekly, coordinated by trained higher-level professionals, among whom nurses play a significant role^(6)^. The sessions can be conducted individually or in groups. Following this, two biweekly maintenance sessions and a monthly maintenance session are recommended until one year is completed^(7)^. However, a portion of individuals who start the treatment ends up abandoning the treatment instead of quitting smoking. Studies conducted in the states of Alagoas in 2021 and Santa Catarina in 2018 found treatment abandonment rates of 34.7% and 40.4%, respectively^(8-9)^. In the southern region of Brazil, a study conducted between 2017 and 2019 revealed abandonment rates that, in some periods, exceeded 60%^(10)^.

The abandonment of treatment by a single individual can result in the non-utilization of up to six hours of interdisciplinary collective care during the intensive phase and approximately 16.5 hours during the maintenance phase. Additionally, each abandonment prevents another person from accessing treatment, depriving them of the opportunity to have their cessation process facilitated by specialized professionals and medications. Optimizing available resources and combating waste are challenges for the sustainability of the Unified Health System (SUS)^(11)^.

Exploring factors that can increase or decrease the risk of treatment abandonment is essential for advancing the fight against smoking and optimizing SUS resources. However, scientific production on the subject is still scarce^(9,12-15)^, and some of this production is becoming outdated^(12,14-15)^. Therefore, we questioned: are the age at which one started smoking, the age at which one began treatment, the number of cigarettes smoked per day, sex, degree of nicotine dependence, and living with a smoker associated with the risk of abandoning smoking treatment during its intensive phase?

## OBJECTIVES

To evaluate the relative risk of treatment abandonment during the intensive phase of smoking cessation.

## METHODS

### Ethical Aspects

The research project was approved by the Research Ethics Committee (REC) with Human Subjects of the School of Nursing of Ribeirão Preto, University of São Paulo and it adhered to the principles of the National Health Council Resolution 466/2012.

When submitting the research project to the REC, a waiver of the Informed Consent Form (ICF) was requested because the study population had been discharged from the health unit for at least three years and contact with them was complicated by the pandemic context. The waiver of the ICF was authorized by the REC, so the research participants were not directly contacted for the signing of the term.

### Study Design, Period, and Location

This is a retrospective cohort study with a quantitative approach, conducted using secondary data, following the recommendations of the Strengthening the Reporting of Observational Studies in Epidemiology (STROBE)^(16)^.

Data collection was conducted from January to April 2023 through consultation of electronic medical records and the smoking treatment schedule for the years established for the research, from 2015 to 2019. For the study, a specialty outpatient clinic of the public health network in Ribeirão Preto, in the interior of the state of São Paulo, was selected. This clinic began offering treatment in 2012 and, being located in a central region, serves people from different areas of the city

### Sample, Inclusion, and Exclusion Criteria

According to the Program for Attention to People with Chronic Diseases, which coordinates smoking cessation treatment actions in the municipality, the average number of users who started smoking cessation treatment during the study period is estimated at 2,000 people. The sample size of the study was calculated using the equation n = (p(1-p)*Z^2)/d^2^(17)^, where:

n is the sample size,

p is the expected proportion (prevalence),

Z is the value from the normal distribution corresponding to the desired confidence level,

d is the margin of error.

To determine the minimum sample size, an abandonment prevalence of 30.7% was assumed^(18)^, with a 95% confidence interval and a margin of error of 0.05, estimating that at least 327 individuals would be needed to investigate the prevalence of treatment abandonment in this population.

Between 2015 and 2019, Ribeirão Preto had five accredited and regularly active smoking cessation treatment teams. In 2015, two teams were working at the outpatient clinic selected for the study. However, in mid-2017, one of the teams was transferred to another unit. During the transfer process of the second team, the name of the professional to whom the users had been assigned was removed from the system, making it impossible to generate the list of people attended by this team, thus preventing the inclusion of data from those treated by this team in the study.

Therefore, the study included data from 396 smokers, aged 18 or older, who underwent smoking cessation treatment with the team that operated continuously at the study site between 2015 and 2019. Data from 19 individuals who underwent treatment but whose information was not recorded in the electronic medical record and could not be retrieved from the physical records were excluded from the study.

### Study Protocol

**Figure 1:**
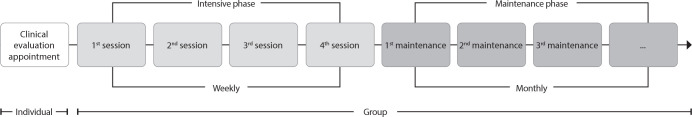
Phases of smoking cessation treatment at the study site, Ribeirão Preto, São Paulo, Brazil, 2023

The study site offers an intensive approach to smoking cessation treatment, structured around an initial individual clinical evaluation, four weekly group sessions (intensive phase), and monthly group sessions (maintenance phase) until the user completes 12 months tobacco-free. Figure 1 presents the adaptation of the treatment model proposed by the Clinical Protocol and Therapeutic Guidelines for Smoking, which is adopted by the unit^(7)^.

### Data Collection

Data were obtained from medical records. The unit uses the acronym AF (Atendimento Fumante, or Smoker’s Care) for intensive smoking cessation treatment appointments. The records marked with the acronym AF were read in full, and the relevant data were extracted and entered into spreadsheets in Excel.

Information collected included: date of the clinical evaluation appointment, date of birth, sex, living with another smoker at home, Fagerström test score, number of cigarettes smoked per day, age at which the individual started smoking regularly, and attendance dates for treatment sessions. Treatment abandonment was defined as failure to attend sessions following the clinical evaluation appointment, including the 1st, 2nd, or 3rd structured treatment sessions. The degree of nicotine dependence was categorized as very low, low, medium, high, and very high according to the classification proposed by the Fagerström nicotine dependence test^(19)^.

### Data Analysis and Statistics

The collected data were entered into an Excel spreadsheet with double entry to minimize potential errors in study variables. Subsequently, the data were coded and classified to be exported to SAS 9.4 statistical software, where they were analyzed using descriptive and inferential statistics.

To analyze the association between the variables of interest and treatment abandonment, estimating the corresponding crude and adjusted Relative Risk, a Poisson regression model with robust variance^(20)^, both simple and multiple, was used. A significance level of 5% was adopted for all analyses.

## RESULTS

Of the 396 (100.0%) individuals who started smoking cessation treatment between 2015 and 2019 at the study site, 109 (27.5%) abandoned the treatment before completing the intensive phase. The average age of those who abandoned the treatment and those who did not was 51.84 (SD=12.59) and 55.39 (SD=10.82) years, respectively. The average number of cigarettes smoked per day was 23.6 (SD=11.98) among those who abandoned the treatment and 23.9 (SD=11.89) among those who did not.

Regarding sex, a similar proportion of women and men abandoned the treatment, as well as a similar proportion of individuals who lived with smokers and those who did not, as shown in Table 1.

**Table 1:** Smoking cessation treatment abandonment according to sex, living with another smoker or not, and degree of nicotine dependence, N=396, Ribeirão Preto, São Paulo, Brazil, 2023

Variable	Treatment Abandonment	
	No (n,%)	Yes (n,%)
Sex		
Female (n=272)	198 (72.79)	74 (27.21)
Male (n=124)	89 (71.77)	35 (28.23)
Living with another smoker		
No (n=251)	184 (73.31)	67 (26.69)
Yes (n=141)	99 (70.21)	42 (29.79)
Degree of nicotine dependence - Fagerström		
Very low (n=12)	7 (58.33)	5 (41.67)
Low (n=37)	23 (62.16)	14 (37.84)
Medium (n=40)	32 (80)	8 (20)
High (n=172)	127 (73.84)	45 (26.16)
Very high (n=132)	95 (71.97)	37 (28.03)

Regarding the timing of abandonment, 24 (6.0%) individuals did so after the clinical evaluation appointment, 37 (9.3%) after the 1st structured session, 30 (7.1%) after the 2nd session, and 18 (4.5%) after the 3rd session. The relative risk of abandoning the treatment did not differ among individuals with different degrees of nicotine dependence, as shown in Table 2.

**Table 2:** Relative risk of smoking cessation treatment abandonment according to different degrees of nicotine dependence, N=396, Ribeirão Preto, São Paulo, Brazil, 2023

Effect	Crude Regression Model			
	Relative Risk (abandonment)	95% CI		*p* value
		LL	UL	
Degree of dependence (very low vs low)	1.10	0.39	3.07	0.99
Degree of dependence (very low vs medium)	2.08	0.61	7.12	0.48
Degree of dependence (very low vs high)	1.59	0.63	4.00	0.64
Degree of dependence (very low vs very high)	1.49	0.58	3.78	0.77
Degree of dependence (low vs medium)	1.89	0.67	5.31	0.44
Degree of dependence (low vs high)	1.45	0.74	2.83	0.56
Degree of dependence (low vs very high)	1.35	0.67	2.70	0.76
Degree of dependence (medium vs high)	0.76	0.31	1.89	0.93
Degree of dependence (medium vs very high)	0.71	0.28	1.83	0.87
Degree of dependence (high vs very high)	0.93	0.55	1.57	0.99

*LL - Lower Limit; UL - Upper Limit; 95% CI: Confidence Interval (95%).*

Living with a smoker in the same house, being male or female, the number of cigarettes smoked, the age at which smoking started, and the Fagerström score conferred a relative risk of abandoning treatment that, in the statistical analysis of this study, was not significant. However, it is estimated that, on average, for each year increase in the participant’s age at the start of treatment, the risk of abandonment decreases by an average of 2% [(1-0.98)*100], as shown in Table 3.

**Table 3:** Relative risk of smoking cessation treatment abandonment, N=396, Ribeirão Preto, São Paulo, Brazil, 2023

Effect	Crude Model (n=396)				Adjusted Model (n=385)			
	Relative Risk (abandonment)	Confidence Interval (95%)		*p* value	Relative Risk (abandonment)	Confidence Interval (95%)		*p* value
Living with a smoker at home (n=392) (Yes vs No)	1.12	0.80	1.56	0.52	1.07	0.76	1.52	0.69
Sex (n=396) (F vs M)	0.96	0.69	1.35	0.83	0.93	0.67	1.31	0.69
Number of cigarettes smoked per day (n=393)	1.00	0.98	1.01	0.82	1.00	0.98	1.01	0.68
Age when started smoking regularly (n=394)	1.00	0.96	1.03	0.83	0.99	0.96	1.03	0.70
Fagerström score (n=393)	0.98	0.90	1.06	0.60	0.99	0.89	1.10	0.84
Age at clinical evaluation appointment (n=396)	0.98	0.97	0.99	<0.01	0.98	0.97	1.00	0.02

## DISCUSSION

The study demonstrated that approximately one in four people who started smoking cessation treatment ended up abandoning the treatment, not the tobacco, during the initial sessions. This finding is concerning and highlights the need to rethink the care provided, focusing on the period before the intensive approach begins and the first few weeks of it. Reducing treatment abandonment can contribute to lowering premature mortality from non-communicable diseases and strengthening the WHO FCTC.

Regarding age, the study showed that for each year increase in age, the risk of abandoning smoking cessation treatment decreased by an average of 2%. This finding corroborates studies by Cabrita et al.^(21)^ conducted in Portugal, and Conroy et al.^(22)^ conducted in the United States, which found that being younger was a predictor for treatment abandonment, and being older was associated with a lower probability of abandonment, respectively. A study by Pawlina et al.^(12)^ conducted in Brazil in 2012 found an association between treatment abandonment and younger age groups. The study supports research that found a higher prevalence of treatment abandonment among younger people, such as Rocha et al.^(9)^, conducted in Santa Catarina in 2018.

Among younger individuals, the prospect of illness resulting from smoking seems distant, leading them to believe they will have other opportunities to quit smoking^(12)^. Between perceived risks and uncertainties regarding the consequences, many people, especially younger ones, find in tobacco an immediate escape valve for everyday problems^(23)^, ignoring the harm caused by such a decision. Aging brings greater clarity and often allows people to begin experiencing the consequences of smoking, at which point there is a greater willingness to continue treatment, as evidenced by the study.

The healthcare model adopted in Brazil is still strongly focused on disease. People tend to take care of themselves only when they already show manifestations of health problems. Health services themselves are also organized from a curative perspective, targeting sick individuals and operating extended hours only for acute conditions (urgent and emergency), while preventive and health promotion activities are carried out at times incompatible with the working hours of most of the Brazilian population. It is understood that these activities could be offered at times that favor worker participation.

State health departments are responsible for training professionals to work in smoking cessation treatment within the framework of the PNCT^(24)^. Revisiting the training process, qualifying professionals to adopt approaches based on participatory methodologies that consider the initial knowledge and experiences of participants and that de-territorialize the established modes of health promotion^(25)^, could help reduce the risk of treatment abandonment in this population, which notoriously has greater difficulty maintaining treatment continuity.

In the present study, statistical analysis did not show an association between the risk of treatment abandonment and the sex of the participants, unlike the study conducted in Portugal in 2018, which found an association between being female and abandoning treatment^(21)^. However, the discrepancy between the number of females (n=272) and males (n=124) who started treatment supports the studies by Costa et al.^(13)^, Santos et al.^(26)^, and Malta et al.^(27)^. This disparity is associated with intersectional factors that influence the supposed difficulty of men taking care of and seeking their own health but also reveals societal changes, such as women’s greater investment in their independence and their insertion into the labor market, where smoking is increasingly seen as undesirable. Therefore, it is necessary to improve the public policy debate on health, considering intersectional factors, and social and cultural vulnerabilities in health practices^(28)^. Numerous factors may have contributed to participants abandoning treatment, including lack of support, resistance to change, psychological and physical dependence, social pressure, ineffective coping strategies, insufficient knowledge about the harms, risk behaviors, and more^(29)^.

A study that analyzed data from health units accredited for smoking cessation treatment by the public health system in the state of São Paulo, between 2012 and 2015, found an average abandonment rate of 30.7%^(18)^. Costa and collaborators^(13)^ conducted a study in Campinas, in the interior of São Paulo, analyzing the issue of smoking cessation treatment abandonment between 2016 and 2018 among 276 people who started treatment and found an abandonment rate of 31.0%. It is noteworthy that in the service studied, the abandonment rate was 27.5%, indicating improved performance compared to the state of São Paulo. A retrospective study conducted in Minas Gerais with data from people who started smoking cessation treatment between 2009 and 2013, whose treatment did not include free access to pharmacotherapy through the SUS, found a treatment abandonment rate of 84.6%^(27)^. This finding reaffirms the significant support that the SUS, as a public, free, and universal health system, provides to a large portion of the Brazilian population^(30)^. The health team at the unit where the study was conducted reported periods of medication shortages for smoking cessation treatment during the analyzed period. However, due to the inability to specify these periods, it was not possible to analyze their potential impact on treatment abandonment rates.

There is a significant expectation among people undergoing smoking cessation treatment regarding the use of medications^(31)^. However, smoking involves not only physical dependence, which is typically treated with medications, but also behavioral and psychological dependencies, for which there are no medications, only behavior change. The behavior change required for smoking cessation is a complex and multifactorial process that demands a comprehensive approach ^(10)^. According to Campos and Gomide^(32)^, “behavior change is not subject to simple and direct information, although it can happen. It is an achievement resulting from endogenous and exogenous elements, extracted from everyday life in society, over time”.

According to Merhy et al.^(25)^, “any concrete reality can become a means of life and health or a crossing that captures us and brings illness and the force of death, depending on the singular existential configurations we need to encounter”. The authors explain the complexity that the arrangements produced by smoking, for example, confer on the health-disease-care process. Here, arrangement is understood as “the encounter between heterogeneous elements for the invention of a new co-functioning”^(25)^, such as when a cigarette becomes a “companion” in dealing with everyday problems. Promoting health in this context involves more than prescribing a behavior change; it involves co-constructing with the smoker other arrangements that produce life. In this study, 37 (9.3%) people abandoned treatment after the 1st structured session. It is noteworthy that in this session, in addition to being confronted with the impossibility of medication treatment for behavioral and psychological dependencies, participants are invited to set a quit date, a moment fraught with strong ambivalence^(33)^, highlighting the importance of investing in the co-construction of other arrangements early in the treatment.

The Fagerström nicotine dependence test, despite some limitations, is a widely used tool for evaluating smokers^(34)^. In this study, it was observed that the proportion of people with very low and low nicotine dependence who abandoned treatment was higher compared to those with medium, high, and very high nicotine dependence. However, as shown in the results, statistical analysis did not show an association between the degree of nicotine dependence and the risk of treatment abandonment. A study conducted by Rocha et al. in Araranguá, Santa Catarina, in 2018 found that people with high nicotine dependence abandoned treatment more frequently^(9)^. It is important to note that, just as no association was found between the degree of nicotine dependence and the risk of treatment abandonment, no association was found between the number of cigarettes smoked per day and the risk of treatment abandonment. This contrasts with the study by Cabrita et al.^(21)^, which found that having a lower tobacco load was a predictor of treatment abandonment.

Social, familial, psychological, behavioral, and environmental factors influence the decision to continue or abandon treatment, as quitting smoking is a complex challenge that leaves many contemplating quitting in personal conflict, requiring mutual efforts not only from the individual but from everyone around them^(35)^. It can be observed that a slightly higher proportion of people living with another smoker abandoned treatment, but statistical analysis did not show an association between living with a smoker and the risk of treatment abandonment, as shown in the results of this research. No studies were found that analyzed this association. However, a study that evaluated conditions associated with success and failure in smoking cessation among people undergoing treatment after dental implant procedures, from 2019 to 2021 in the state of São Paulo, observed that living or working with smokers is not a predictive factor of treatment success or failure in this population^(36)^.

The findings of this research raise questions about how smoking cessation treatment is offered to the younger adult population. It is considered that investments in expanding the availability of treatment times could help reduce treatment abandonment and that educational actions based on participatory models and pedagogical references that consider the learners’ reality could be used, in addition to implementing pre-treatment interventions to reduce the risk of abandonment. The development of new research exploring the phenomenon of treatment abandonment from more comprehensive approaches could strengthen the WHO FCTC and reduce premature mortality from non-communicable chronic diseases, aligning with targets 3.4 and 3.a of Sustainable Development Goal 3 of the 2030 Agenda.

### Study limitations

This research has limitations. Firstly, it was developed solely from secondary data, without direct input from the individuals involved, and did not address social, economic, and cultural aspects that could affect the outcome. Secondly, despite having a significant number of participants, the study was conducted using data from individuals who started treatment with a single team, which may compromise its external validity. Additionally, the number of people with very low and low levels of dependence who were part of this study population was small. Future studies comparing treatment abandonment rates among people with different levels of nicotine dependence, using more balanced samples concerning dependence levels, could help better elucidate the phenomenon.

### Contributions to the Field of Nursing, Health, or Public Policy

Although Brazil has made progress in combating smoking and is internationally recognized for its efforts, approximately 20 million Brazilians still smoke^(27,37)^. Smoking cessation is important at any stage of life. However, early cessation significantly reduces the risk of developing complications and mortality^(38)^. The finding that the relative risk of abandoning smoking cessation treatment during its intensive phase is higher among younger individuals is evidence that can support planning actions to encourage treatment continuity, especially among younger people.

Nursing plays an essential role in tobacco control and is therefore engaged in achieving the SDGs through teaching, research, management, and care. The results of this study update the scientific evidence on the subject.

## CONCLUSIONS

It was observed that the risk of abandoning smoking cessation treatment during its intensive phase decreases as the age of the person in treatment increases. It is necessary to invest in actions that encourage the continuity of treatment, especially among younger adults.

The third goal of the SDGs in the 2030 Agenda is to ensure healthy lives and promote well-being for all at all ages. The findings of this study question whether the care offered to people who wish to quit smoking effectively covers individuals of all age groups and invite managers and treatment teams to rethink the conditions of access to smoking cessation treatment for younger people. It is also important to revisit the pedagogical strategies adopted, considering the technological evolution we are experiencing and incorporating scientific advancements on the subject.

The number of cigarettes smoked per day, the age at which one starts smoking, sex, and living with a smoker do not seem to significantly influence the risk of abandoning smoking cessation treatment. Thus, social, cultural, political, and economic aspects need to be investigated to gain a better understanding of smoking cessation treatment abandonment and to provide more personalized care.
