# Supplementary material for: Risk of smoking cessation treatment dropout: a cohort to help (re)think care
Source: Rev Bras Enferm. 2024 Aug 30;77(Suppl 2):e20230537. doi: 10.1590/0034-7167-2023-0537 (PMC11370772; doi:10.1590/0034-7167-2023-0537)
Supplement: Translation

# Translation: Risco de abandono do tratamento do tabagismo: uma coorte para ajudar a (re)pensar o cuidado

**DOI:** 10.1590/0034-7167-2023-0537pt

**Authors:** Janaina Pereira da Silva, Adriana Ignácio de Pádua, Ruan Víctor dos Santos Silva, Felipe Lima dos Santos, Poliana Silva de Oliveira, Priscila Norié Araujo-Betetti, Joris Thievenaz, Cinira Magali Fortuna

**Affiliations:** I Universidade de São Paulo. Ribeirão Preto, São Paulo, Brasil; II Université Paris Est-Créteil. Créteil, Île de France, França; III Universidade de São Paulo, Hospital das Clínicas. Ribeirão Preto, São Paulo, Brasil; IV CY Cergy Paris Université. Gennevilliers, Île de France, França

**Keywords:** Tabagismo, Controle do Tabaco, Pacientes Desistentes do Tratamento, Saúde Pública, Enfermagem em Saúde Pública

## Abstract

**Objetivos:**

avaliar o risco relativo de abandono do tratamento do tabagismo durante sua fase intensiva.

**Métodos:**

estudo de coorte retrospectivo e quantitativo, desenvolvido a partir da consulta aos prontuários eletrônicos de pessoas que iniciaram o tratamento do tabagismo entre os anos de 2015 e 2019 em um ambulatório de especialidades de uma cidade do interior de São Paulo, Brasil. O risco relativo de abandonar o tratamento foi calculado utilizando o modelo de regressão de Poisson.

**Resultados:**

observou-se que, das 396 (100,0%) pessoas que iniciaram o tratamento, 109 (27,5%) o abandonaram antes do término da fase intensiva. A cada um ano de aumento na idade, o risco de abandono do tratamento do tabagismo diminuiu, em média, 2%.

**Conclusões:**

o risco de abandono do tratamento do tabagismo é maior em pessoas mais jovens. Faz-se necessário repensar o cuidado ofertado a adultos mais jovens, de forma a favorecer a continuidade do tratamento.

## INTRODUÇÃO

A Agenda 2030 é uma iniciativa da Organização das Nações Unidas (ONU) que propõe o alcance de 17 Objetivos de Desenvolvimento Sustentável (ODS), compondo um plano de ação para as pessoas, o planeta e a prosperidade por meio de 169 metas. O objetivo número 3 da Agenda é “assegurar uma vida saudável e promover o bem-estar para todos, em todas as idades”. Este objetivo é composto por 13 metas, sendo duas delas relacionadas ao tabagismo: “fortalecer a implementação da Convenção-Quadro para o Controle do Tabaco da Organização Mundial da Saúde (CQCT/OMS) em todos os países” e “reduzir em um terço a mortalidade prematura por doenças não transmissíveis via prevenção e tratamento, e promover saúde mental e bem-estar”^(1)^.

O tabagismo é um problema de saúde pública que impacta negativamente a saúde da população pela sua relação direta com o desenvolvimento de doenças cardiovasculares, pulmonares e oncológicas^(2)^. A pessoa nesta condição soma prejuízos para si (gastos com a compra de cigarro, tratamento de doenças ocasionadas pelo ato de fumar, inalação da fumaça do tabaco), bem como para as pessoas que estão ao seu redor (fumantes passivos).

No Brasil, a Política Nacional de Controle do Tabaco (PNCT) é o eixo condutor da CQCT/OMS. Apesar de ameaçada pela interferência da cadeia produtiva, a PNCT conseguiu estabelecer medidas de grande impacto, como o aumento dos impostos e dos preços dos produtos derivados do tabaco e a restrição de peças publicitárias que incentivam seu uso^(3)^, fazendo com que a prevalência estimada de tabagistas no Brasil fosse reduzida de 18,2% em 2008 para 12,6% em 2019^(4)^. Enquanto muitos países ainda estão no início do desenvolvimento de medidas voltadas ao controle do tabagismo, o Brasil se dedica à expansão de sua rede de tratamento e à consolidação de sua linha de cuidado^(5)^.

O Artigo 14 da CQCT/OMS insta a implementação de medidas para favorecer o abandono do consumo do tabaco, entre elas o tratamento do tabagismo. A abordagem intensiva para o tratamento do tabagismo consiste no aconselhamento terapêutico estruturado e pode ou não incluir a terapia medicamentosa. Nela estão previstas a realização de uma consulta de avaliação clínica individual e quatro sessões estruturadas, preferencialmente semanais, coordenadas por profissionais de nível superior capacitados, dentre os quais os enfermeiros vêm desempenhando um papel relevante^(6)^. As sessões podem ser desenvolvidas de forma individual ou em grupo. Na sequência, está preconizada a realização de duas sessões quinzenais de manutenção seguidas de uma sessão mensal de manutenção até completar um ano^(7)^. Entretanto, uma parte das pessoas que inicia o tratamento, ao invés de abandonar o tabagismo, acaba abandonando o tratamento. Estudos realizados nos estados de Alagoas no ano de 2021 e de Santa Catarina no ano de 2018, encontraram percentuais de abandono do tratamento de 34,7% e 40,4%, respectivamente^(8-9)^. Na região sul do Brasil, estudo realizado entre os anos 2017 e 2019 revelou percentagem de abandono que chegou a ser, em alguns períodos, superior a 60%^(10)^.

O abandono do tratamento por um único indivíduo pode resultar no não aproveitamento de até seis horas de atendimento coletivo interdisciplinar durante a fase intensiva e de aproximadamente 16,5 horas durante a fase de manutenção. Além disso, cada abandono inviabiliza o acesso de outra pessoa ao tratamento, subtraindo desta a oportunidade de ter o processo de cessação facilitado por profissionais especializados e por medicamentos. A otimização dos recursos disponíveis e o combate ao desperdício são desafios para a sustentabilidade do Sistema Único de Saúde (SUS)^(11)^.

A exploração de fatores que podem aumentar ou diminuir o risco de abandono do tratamento é imprescindível para o avanço no enfrentamento do tabagismo e para a otimização dos recursos do SUS. No entanto, a produção científica sobre o tema ainda é escassa^(9,12-15)^, e parte desta produção começa a tornar-se antiga^(12,14-15)^. Desse modo, questionou-se: a idade em que começou a fumar, a idade em que iniciou o tratamento, a quantidade de cigarros fumados por dia, o sexo, o grau de dependência à nicotina e morar ou não com uma pessoa tabagista estão associados ao risco de abandono do tratamento do tabagismo em sua fase intensiva?

## OBJETIVOS

Avaliar o risco relativo de abandono do tratamento do tabagismo durante sua fase intensiva.

## MÉTODOS

### Aspectos Éticos

O projeto de pesquisa foi aprovado pelo Comitê de Ética em Pesquisa (CEP) com Seres Humanos da Escola de Enfermagem de Ribeirão Preto da Universidade de São Paulo e respeitou os fundamentos da Resolução do Conselho Nacional de Saúde 466/2012.

Na submissão do projeto de pesquisa ao CEP, foi solicitada a dispensa do Termo de Consentimento Livre e Esclarecido (TCLE), dado que a população do estudo já havia recebido alta da unidade de saúde há pelo menos três anos e o contato com esta foi dificultado pelo contexto pandêmico. A dispensa do TCLE foi autorizada pelo CEP, de modo que os participantes da pesquisa não foram acessados diretamente para assinatura do termo.

### Desenho, Período e Local de Estudo

Trata-se de um estudo de abordagem quantitativa do tipo coorte retrospectivo, realizado a partir de dados secundários, que seguiu as recomendações do *STrengthening the Reporting of OBservational studies in Epidemiology* (STROBE)^(16)^.

A coleta de dados foi realizada de janeiro a abril de 2023 por meio de consulta ao prontuário eletrônico e ao cronograma do tratamento do tabagismo dos anos estabelecidos para a pesquisa, de 2015 a 2019. Para a realização do estudo, foi selecionado um ambulatório de especialidades da rede pública de saúde de Ribeirão Preto, interior do estado de São Paulo, que começou a oferecer o tratamento em 2012 e, por estar localizado em uma região central, recebe pessoas de diferentes regiões da cidade.

### Amostra, Critérios de Inclusão e Exclusão

De acordo com o Programa de Atenção às Pessoas com Doenças Crônicas, que coordena as ações de tratamento do tabagismo no município, a média de usuários que iniciaram o tratamento do tabagismo no período do estudo é estimada em 2000 pessoas. O tamanho da amostra do estudo foi calculado através da equação n = (p(1-p)*Z^2)/d^2^(17)^, onde:

n é o tamanho da amostra,

p é a proporção esperada (prevalência),

Z é o valor da distribuição normal correspondente ao nível de confiança desejado,

d é a margem de erro.

Para determinar o tamanho mínimo da amostra, assumiu-se uma prevalência de abandono de 30,7%^(18)^, um intervalo de confiança de 95% e uma margem de erro de 0,05, estimando-se que seriam necessários no mínimo 327 indivíduos para investigar a prevalência de abandono do tratamento do tabagismo nesta população.

Entre 2015 e 2019, Ribeirão Preto contava com cinco equipes de tratamento do tabagismo credenciadas e em atividade regular. Em 2015, duas equipes trabalhavam no ambulatório selecionado para a realização do estudo. Entretanto, em meados de 2017, uma das equipes foi transferida de unidade. No processo de transferência da segunda equipe, o nome da profissional para quem os usuários haviam sido agendados foi retirado do sistema, impossibilitando a geração da lista de pessoas atendidas por esta equipe, fato que inviabilizou a inclusão dos dados referentes às pessoas que realizaram o tratamento junto a esta equipe no estudo.

Desse modo, foram incluídos no estudo os dados referentes a 396 pessoas tabagistas, com idade igual ou superior a 18 anos, que realizaram o tratamento para cessação do tabagismo com a equipe que atuou no local do estudo ininterruptamente entre os anos de 2015 e 2019. Foram excluídos do estudo os dados referentes a 19 pessoas que realizaram o tratamento, mas cujos dados não estavam registrados no prontuário eletrônico e não puderam ser resgatados pelo acesso ao prontuário físico.

### Protocolo do estudo

O local do estudo oferece a abordagem intensiva para o tratamento do tabagismo, com tratamento estruturado em uma consulta de avaliação clínica individual, quatro sessões grupais semanais (fase intensiva) e sessões grupais mensais (fase de manutenção) até que o usuário complete 12 meses livre do tabaco. A Figura 1 apresenta a adaptação do modelo de tratamento proposto pelo Protocolo Clínico e Diretrizes Terapêuticas do Tabagismo, que é adotada pela unidade^(7)^.

**Figura 1:** Fases do tratamento do tabagismo no local do estudo, Ribeirão Preto, São Paulo, Brasil, 2023

### Coleta de Dados

Os dados foram obtidos a partir da leitura dos prontuários. A unidade adota a sigla AF (Atendimento Fumante) para os atendimentos da abordagem intensiva para o tratamento do tabagismo. Os atendimentos registrados utilizando a sigla AF foram lidos na íntegra, e os dados de interesse foram extraídos e inseridos em planilhas no programa *Excel*.

Foram coletadas informações como: data da consulta de avaliação clínica, data de nascimento, sexo, residência com outro fumante em casa, valor do teste de Fagerström, quantidade de cigarros fumados por dia, idade em que começou a fumar regularmente e datas de comparecimento às sessões de tratamento. O desfecho abandono foi caracterizado pelo não comparecimento às sessões subsequentes à consulta de avaliação clínica, 1ª, 2ª ou 3ª sessões estruturadas do tratamento. A classificação do grau de dependência à nicotina foi categorizada em muito leve, leve, médio, alto e muito alto, de acordo com a classificação proposta pelo teste de dependência nicotínica de Fagerström^(19)^.

### Análise dos Resultados e Estatística

Os dados coletados foram inseridos em uma planilha Excel com digitação dupla para diminuir possíveis erros das variáveis do estudo. Em seguida, foram codificados e classificados para serem exportados para o *software* estatístico SAS 9.4, onde foram analisados por meio de estatística descritiva e inferencial.

Para a análise da associação entre as variáveis de interesse e o abandono, estimando o Risco Relativo bruto e ajustado correspondente, foi utilizado o modelo de regressão de Poisson com variância robusta^(20)^, simples e múltiplo. Para todas as análises, foi adotado um nível de significância de 5%.

## RESULTADOS

Das 396 (100,0%) pessoas que iniciaram o tratamento do tabagismo entre os anos de 2015 e 2019 no local de estudo, 109 (27,5%) abandonaram o tratamento antes do término da fase intensiva. A média de idade das pessoas que abandonaram e das que não abandonaram o tratamento foi de 51,84 (DP=12,59) e 55,39 (DP=10,82) anos, respectivamente. A quantidade média de cigarros fumados por dia foi de 23,6 (DP=11,98) entre as pessoas que abandonaram o tratamento e de 23,9 (DP=11,89) entre as que não abandonaram.

Em relação ao sexo, uma proporção bastante próxima de mulheres e homens abandonaram o tratamento, assim como uma proporção semelhante de pessoas que residiam ou não com tabagistas, conforme pode ser observado na Tabela 1.

**Tabela 1:** Abandono do tratamento do tabagismo de acordo com o sexo, condição de morar ou não com outro fumante e grau de dependência a nicotina, N=396, Ribeirão Preto, São Paulo, Brasil, 2023

Variável	Abandono do tratamento	
	Não (n,%)	Sim (n,%)
Sexo		
Feminino (n=272)	198 (72,79)	74 (27,21)
Masculino (n=124)	89 (71,77)	35 (28,23)
Mora com outro fumante		
Não (n=251)	184 (73,31)	67 (26,69)
Sim (n=141)	99 (70,21)	42 (29,79)
Grau de dependência à nicotina - Fagerström		
Muito baixo (n=12)	7 (58,33)	5 (41,67)
Baixo (n=37)	23 (62,16)	14 (37,84)
Médio (n=40)	32 (80)	8 (20)
Elevado (n=172)	127 (73,84)	45 (26,16)
Muito elevado (n=132)	95 (71,97)	37 (28,03)

Em relação ao momento em que o abandono ocorreu, 24 (6,0%) pessoas o fizeram após a consulta de avaliação clínica, 37 (9,3%) após a participação na 1ª sessão estruturada, 30 (7,1%) após a 2ª sessão e 18 (4,5%) após a 3ª sessão.

O risco relativo de abandonar o tratamento não diferiu entre as pessoas que possuíam diferentes graus de dependência à nicotina, conforme apresentado na Tabela 2.

**Tabela 2:** Risco relativo de abandono do tratamento do tabagismo de acordo com os diferentes graus de dependência à nicotina, N=396, Ribeirão Preto, São Paulo, Brasil, 2023

Efeito	Modelo de regressão bruto			
	Risco relativo (abandono)	IC95%		Valor de *p*
		LI	LS	
Grau de dependência (muito baixa vs baixa)	1,10	0,39	3,07	0,99
Grau de dependência (muito baixa vs média)	2,08	0,61	7,12	0,48
Grau de dependência (muito baixa vs elevada)	1,59	0,63	4,00	0,64
Grau de dependência (muito baixa vs muito elevada)	1,49	0,58	3,78	0,77
Grau de dependência (baixa vs média)	1,89	0,67	5,31	0,44
Grau de dependência (baixa vs elevada)	1,45	0,74	2,83	0,56
Grau de dependência (baixa vs muito elevada)	1,35	0,67	2,70	0,76
Grau de dependência (média vs elevada)	0,76	0,31	1,89	0,93
Grau de dependência (média vs muito elevada)	0,71	0,28	1,83	0,87
Grau de dependência (elevada vs muito elevada)	0,93	0,55	1,57	0,99

*LI - Limite Inferior; LS - Limite superior; IC95% - Intervalo de confiança em 95%.*

Conviver ou não com um fumante na mesma casa, ser do sexo feminino ou masculino, a quantidade de cigarros fumados, a idade de início do tabagismo e o valor de Fagerström conferiram um risco relativo de abandonar o tratamento que, na análise estatística deste estudo, não foi relevante. Entretanto, estima-se que a cada um ano de aumento na idade do participante no início do tratamento, o risco de abandono diminui, em média, em 2% [(1-0,98)*100], conforme pode ser observado na Tabela 3.

**Tabela 3:** Risco relativo de abandono do tratamento do tabagismo, N=396, Ribeirão Preto, São Paulo, Brasil, 2023

Efeito	Modelo Bruto(n=396)				Modelo Ajustado (n=385)			
	Risco relativo (abandono)	Intervalo de confiança (95%)		Valor de *p*	Risco relativo (abandono)	Intervalo de confiança (95%)		Valor de *p*
Morar com fumante em casa (n=392) (Sim vs Não)	1,12	0,80	1,56	0,52	1,07	0,76	1,52	0,69
Sexo (n=396) (F vs M)	0,96	0,69	1,35	0,83	0,93	0,67	1,31	0,69
Quantidade de cigarros fumados por dia (n=393)	1,00	0,98	1,01	0,82	1,00	0,98	1,01	0,68
Idade que começou a fumar regularmente (n=394)	1,00	0,96	1,03	0,83	0,99	0,96	1,03	0,70
Valor de Fagerström (n=393)	0,98	0,90	1,06	0,60	0,99	0,89	1,10	0,84
Idade na consulta de avaliação clínica (n=396)	0,98	0,97	0,99	<0,01	0,98	0,97	1,00	0,02

## DISCUSSÃO

O estudo demonstrou que cerca de aproximadamente uma em cada quatro pessoas que iniciaram o tratamento para parar de fumar acabaram abandonando o tratamento, e não o tabaco, ainda nas sessões iniciais. Este achado é preocupante e aponta para a necessidade de repensar o cuidado prestado, com foco no período que antecede o início da abordagem intensiva e nas primeiras semanas desta. Entende-se que a diminuição do abandono do tratamento pode colaborar com a redução da mortalidade prematura por doenças não transmissíveis e com o fortalecimento da CQCT/OMS.

Em relação à idade, o estudo evidenciou que a cada um ano de aumento na idade, o risco de abandono do tratamento do tabagismo diminuiu, em média, em 2%. Este dado corrobora os estudos de Cabrita et al.^(21)^ realizado em Portugal, e de Conroy et al.^(22)^ realizado nos Estados Unidos, que encontraram que ser mais jovem era fator preditor para o abandono do tratamento e ter idade mais elevada estava relacionada com a menor probabilidade de abandono, respectivamente. Estudo realizado por Pawlina et al.^(12)^ no Brasil no ano de 2012 encontrou associação entre abandono do tratamento e menor faixa etária. O estudo ratifica pesquisas que encontraram maior prevalência de abandono do tratamento entre pessoas mais jovens, como o de Rocha et al.^(9)^, realizado em Santa Catarina em 2018.

Entre pessoas mais jovens, a perspectiva de adoecimento decorrente do tabagismo parece algo muito distante, o que lhes faz acreditar que terão outras oportunidades para parar de fumar^(12)^. Entre riscos percebidos e incertezas em relação às consequências, muitas pessoas, especialmente as mais jovens, encontram no tabaco uma válvula de escape imediata para problemas do cotidiano^(23)^, ignorando os prejuízos advindos de tal decisão. O avanço da idade confere maior lucidez e, muitas vezes, permite que as pessoas comecem a experimentar as consequências do tabagismo, momento em que revela-se uma maior disposição para a continuidade do tratamento, conforme evidenciado pelo estudo.

O modelo de atenção à saúde adotado no Brasil ainda é fortemente voltado para a doença. As pessoas tendem a se cuidar apenas quando já apresentam manifestações de problemas de saúde. Os próprios serviços de saúde também se organizam a partir de uma perspectiva curativista, tendo como público alvo as pessoas doentes, funcionando com horários de atendimento estendido apenas para condições agudas (urgência e emergência), sendo as atividades preventivas e de promoção de saúde realizadas em horários incompatíveis com as jornadas de trabalho da maior parte da população brasileira. Entende-se que as atividades poderiam ser oferecidas em horários que favorecessem a participação de trabalhadores.

As secretarias estaduais de saúde são responsáveis pela formação dos profissionais para a atuação no tratamento do tabagismo no âmbito da PNCT^(24)^. Considera-se que revisitar o processo formativo, qualificando os profissionais para a adoção de abordagens pautadas em metodologias participativas, que considerem os saberes iniciais e experiências dos participantes e que desterritorializem os modos instituídos de promoção da saúde^(25)^, poderia colaborar com a diminuição do risco de abandono do tratamento nesta população que, notoriamente, apresenta maior dificuldade em manter a continuidade do tratamento.

No presente estudo, a análise estatística não evidenciou associação entre o risco de abandono do tratamento e o sexo dos participantes, diferente do estudo realizado em Portugal no ano de 2018, que encontrou uma associação entre ser do sexo feminino e abandonar o tratamento^(21)^. Contudo, a discrepância entre o número de pessoas que iniciaram o tratamento do sexo feminino (n=272) e masculino (n=124) corrobora os estudos de Costa et al.^(13)^, Santos et al.^(26)^ e Malta et al.^(27)^. Esta disparidade se associa a fatores interseccionais que influenciam na suposta dificuldade de homens cuidarem e buscarem pela própria saúde, mas também pode revelar mudanças na sociedade, como o maior investimento das mulheres em sua independência e sua inserção no mercado de trabalho, onde fumar vem sendo considerado uma característica não desejável. Faz-se assim necessário aprimorar, no âmbito das políticas públicas, o debate acerca da saúde, considerando fatores interseccionais, vulnerabilidades sociais e culturais nas práticas de saúde^(28)^. Inúmeros fatores podem ter contribuído para que os participantes abandonassem o tratamento, entre estes: falta de suporte, resistência a mudanças, dependência psicológica e física, pressão social, estratégias de enfrentamento ineficazes, conhecimento insuficiente sobre os danos, comportamento de risco, entre outros^(29)^.

Estudo que analisou dados de unidades de saúde credenciadas para o tratamento do tabagismo pelo sistema público de saúde no estado de São Paulo, entre os anos de 2012 e 2015, encontrou um percentual médio de abandono de 30,7%^(18)^. Costa e colaboradores^(13)^ desenvolveram um estudo em Campinas, interior do estado de São Paulo, analisando a questão do abandono do tratamento do tabagismo entre os anos de 2016 e 2018 junto a 276 pessoas que iniciaram o tratamento e encontraram uma percentagem de abandono de 31,0%. Destaca-se que, no serviço estudado, a taxa de abandono foi de 27,5%, apontando para uma melhora de desempenho em relação ao estado de São Paulo. Já um estudo retrospectivo, realizado em Minas Gerais, com dados referentes a pessoas que iniciaram o tratamento do tabagismo entre 2009 e 2013, cujo tratamento não previa o acesso à terapia medicamentosa de forma gratuita pelo SUS, encontrou uma taxa de abandono do tratamento de 84,6%^(27)^. Este achado reafirma a relevância assistencial que o SUS, enquanto um sistema público, gratuito e universal, tem para grande parte da população brasileira^(30)^. A equipe de saúde da unidade onde o estudo foi realizado relatou períodos de desabastecimento de medicamentos para o tratamento do tabagismo no período analisado. Entretanto, dada a impossibilidade de especificar esses períodos, não foi possível analisar seus possíveis impactos nas taxas de abandono do tratamento.

Há uma expectativa significativa das pessoas que realizam o tratamento do tabagismo em relação ao uso dos medicamentos^(31)^. Entretanto, o tabagismo envolve, além da dependência física, que é habitualmente tratada com medicamentos, as dependências comportamental e psicológica, para as quais não existe medicamento, mas sim mudança de comportamento. A mudança de comportamento necessária para a cessação do tabagismo é um processo complexo e multifatorial, que demanda uma abordagem ampliada^(10)^. Para Campos e Gomide^(32)^, “...a mudança de comportamento não está sujeita à informação simples e direta, embora aconteça. Ela é conquista resultante de elementos endógenos e exógenos, extraídos do cotidiano em sociedade, ao longo do tempo”.

De acordo com Merhy et al.^(25)^, “qualquer realidade concreta pode vir a ser um agenciamento de vida e saúde ou um atravessamento que nos captura e traz adoecimento e força de morte, a depender de configurações existenciais singulares com as quais precisamos nos encontrar”. Os autores explicitam a complexidade que os agenciamentos produzidos pelo cigarro, por exemplo, conferem ao processo saúde-doença-cuidado. Entende-se aqui por agenciamento “o encontro entre elementos heterogêneos para invenção de um novo cofuncionamento”^(25)^, como quando o cigarro passa a ser utilizado como um “companheiro” no processo de enfrentamento dos problemas do cotidiano. Promover saúde nesse contexto implica mais do que prescrever uma mudança de comportamento, mas co-construir com a pessoa que fuma outros agenciamentos, dessa vez produtores de vida. Neste estudo, a 1ª sessão estruturada foi seguida do abandono do tratamento de 37 (9,3%) pessoas. Destaca-se que nesta sessão, além de serem confrontados com a impossibilidade de tratamento medicamentoso para as dependências comportamental e psicológica, os participantes são convidados a marcar uma data para parar de fumar, e esse momento é atravessado por um forte sentimento de ambivalência^(33)^, reiterando a importância de investir na coconstrução de outros agenciamentos de forma precoce no tratamento.

O teste de dependência nicotínica de Fagerström, apesar de apresentar algumas limitações, é uma ferramenta amplamente utilizada na avaliação de pessoas que fumam^(34)^. Neste estudo, pode-se observar que a proporção de pessoas com grau de dependência à nicotina classificado como muito baixo e baixo que abandonou o tratamento foi maior em relação à proporção de pessoas que possuía grau de dependência à nicotina classificado como médio, elevado e muito elevado. Contudo, conforme pode ser observado nos resultados, a análise estatística não evidenciou associação entre o grau de dependência à nicotina e o risco de abandono do tratamento. Estudo realizado em 2018 por Rocha et al. em Araranguá, Santa Catarina, encontrou que pessoas com dependência elevada à nicotina abandonam mais o tratamento^(9)^. Ressalta-se que, assim como não foi encontrada associação entre o grau de dependência à nicotina e o risco de abandono do tratamento, não foi encontrada associação entre o número de cigarros fumados por dia e o risco de abandono do tratamento. Diferente do estudo de Cabrita et al.^(21)^, em que ter uma menor carga tabágica foi preditor de abandono de tratamento.

Fatores sociais, familiares, psicológicos, comportamentais e ambientais perpassam a tomada de decisão em continuar ou abandonar o tratamento, pois parar de fumar é um desafio complexo que deixa muitos daqueles que consideram a possibilidade de parar em conflito pessoal, necessitando de esforços mútuos não apenas do indivíduo com a condição, mas de todos que o cercam^(35)^. É possível observar que uma proporção discretamente maior de pessoas que moravam com outro fumante abandonou o tratamento, a análise estatística não evidenciou associação entre morar ou não com uma pessoa tabagista e o risco de abandono do tratamento, conforme pode ser verificado nos resultados apresentados nesta pesquisa. Não foram encontrados estudos que analisassem essa associação. Entretanto, um estudo que avaliou condições associadas ao sucesso e fracasso na cessação do tabagismo, entre pessoas submetidas ao tratamento após a realização de implante dentário, no período de 2019 a 2021, no estado de São Paulo, observou que residir ou trabalhar com pessoas tabagistas não é fator preditivo de sucesso ou fracasso do tratamento nesta população^(36)^.

Os achados desta pesquisa instigam questionamentos que tensionam os moldes como o tratamento do tabagismo tem sido ofertado para a população adulta mais jovem. Considera-se que investimentos na ampliação da oferta de horários de atendimento poderiam colaborar para a diminuição do abandono do tratamento e que ações educativas pautadas em modelos participativos e referenciais pedagógicos que considerem a realidade dos aprendizes poderiam ser utilizados, além da implementação de intervenções pré-tratamento para diminuir o risco de abandono. O desenvolvimento de novas pesquisas que explorem o fenômeno do abandono do tratamento, a partir de abordagens mais compreensivas, pode colaborar com o fortalecimento da CQCT/OMS e com a diminuição da mortalidade prematura por doenças crônicas não transmissíveis, metas 3.4 e 3.a do objetivo número 3 dos ODS da Agenda 2030.

### Limitações do estudo

Essa pesquisa possui limitações. Em primeiro lugar, destaca-se o fato de ter sido desenvolvida apenas a partir de dados secundários, sem a escuta direta das pessoas, e de não ter abordado aspectos sociais, econômicos e culturais que poderiam afetar o desfecho. Em segundo lugar, apesar de contar com um número expressivo de participantes, o estudo foi realizado com dados referentes a pessoas que iniciaram o tratamento junto a uma única equipe, o que pode comprometer sua validade externa.

Além disso, o número de pessoas com grau de dependência muito baixo e baixo que integraram a população deste estudo foi pequeno. Estudos futuros que comparem a taxa de abandono do tratamento entre pessoas com diferentes graus de dependência à nicotina, a partir de amostras mais equilibradas em relação ao grau de dependência, poderiam colaborar para uma melhor elucidação do fenômeno.

### Contribuições para área da Enfermagem, Saúde ou Política Pública

Embora o Brasil tenha avançado no enfrentamento do tabagismo e seja reconhecido internacionalmente pelo trabalho que desenvolve, aproximadamente 20 milhões de brasileiros ainda fumam^(27,37)^. A cessação do tabagismo é importante em qualquer fase da vida. Entretanto, a cessação precoce produz uma redução significativa no risco de desenvolvimento de complicações e de morte^(38)^. A constatação de que o risco relativo de abandonar o tratamento do tabagismo em sua fase intensiva é maior quanto menor for a idade da pessoa em tratamento é uma evidência que pode subsidiar o planejamento de ações para favorecer a continuidade do tratamento, especialmente entre pessoas mais jovens.

A enfermagem tem um papel essencial no controle do tabagismo e, por isso, engaja-se no alcance dos ODS por meio do ensino, da pesquisa, da gestão e da assistência. Os resultados deste estudo atualizam as evidências científicas sobre o tema.

## CONCLUSÕES

Observou-se que o risco de abandono do tratamento do tabagismo em sua fase intensiva diminui conforme a idade da pessoa em tratamento aumenta. É preciso investir em ações que favoreçam a continuidade do tratamento, especialmente entre adultos mais jovens.

O objetivo número 3 dos ODS da Agenda 2030 é assegurar uma vida saudável e bem-estar para todos, em todas as idades. Os achados deste estudo questionam se o cuidado ofertado às pessoas que desejam parar de fumar contempla, efetivamente, pessoas de todas as faixas etárias e convidam os gestores e as equipes de tratamento a repensar as condições de acesso ao tratamento do tabagismo junto às pessoas mais jovens. Faz-se importante também revisitar as estratégias pedagógicas adotadas, considerando a evolução tecnológica que vivenciamos e incorporando os avanços da ciência sobre o tema.

A quantidade de cigarros fumados por dia, a idade em que se começa a fumar, o sexo e morar ou não com uma pessoa tabagista parecem não influenciar de forma significativa o risco de abandono do tratamento do tabagismo. Desse modo, aspectos sociais, culturais, políticos e econômicos precisam ser investigados para uma maior compreensão do abandono do tratamento do tabagismo e a realização de um cuidado mais singularizado.
